# Sonorheometry Device Thresholds in Liver Transplantation: An Observational Retrospective Study

**DOI:** 10.3390/jcm13030696

**Published:** 2024-01-25

**Authors:** Maxim Soucy-Proulx, Hiromi Kato, Sean Coeckelenbergh, Salima Naili Kortaia, Laurence Herboulier, Gabriella Pittau, Patrick Pham, Antoinette Lemoine, Jacques Duranteau, Stéphanie Roullet

**Affiliations:** 1 Service d’Anesthésie-Réanimation, Hôpital Paul Brousse, Assistance Publique—Hôpitaux de Paris (AP-HP), Université Paris Saclay, 94800 Villejuif, France; maxim.soucy-proulx@umontreal.ca (M.S.-P.); mynameishiromikato@hotmail.com (H.K.); sean.coeckelenbergh@aphp.fr (S.C.); salima.nailikortaia@aphp.fr (S.N.K.); laurence.herboulier@aphp.fr (L.H.); 2 Department of Anesthesiology, Centre Hospitalier de l’Université de Montréal, Montreal, QC H2X 0A9, Canada; 3 Outcomes Research Consortium, Cleveland, OH 44195, USA; 4 Centre Hépato-Biliaire, Hôpital Paul Brousse, Assistance Publique—Hôpitaux de Paris (AP-HP), Université Paris Saclay, 94800 Villejuif, France; gabriella.pittau@aphp.fr; 5 Service de Biochimie et Oncogénétique, Hôpital Paul Brousse, Assistance Publique—Hôpitaux de Paris (AP-HP), Université Paris Saclay, 94800 Villejuif, France; patrick.pham@aphp.fr (P.P.); antoinette.lemoine@aphp.fr (A.L.); 6 Department of Anesthesiology and Intensive Care, Bicêtre Hospital, Assistance Publique Hôpitaux de Paris (AP-HP), Université Paris Saclay, 94276 Le Kremlin-Bicêtre, France; jacques.duranteau@aphp.fr; 7 INSERM, Hémostase Inflammation Thrombose HITH U1176, Université Paris Saclay, 94276 Le Kremlin-Bicêtre, France

**Keywords:** liver transplantation, point-of-care device, sonorheometry, haemostasis, viscoelastic tests

## Abstract

Background: Liver transplantation (LT) remains a potentially haemorrhagic procedure whose perioperative bleeding and transfusion could be better monitored using point-of-care devices. Quantra^®^ is a device based on sonorheometry to assess whole blood clot formation. Our aims were to describe Quantra^®^ parameters during LT and to study their correlations with standard laboratory parameters, and to determine Quantra^®^ cut-off values for thrombocytopenia, hypofibrinogenemia and coagulation factors’ deficit. Methods: In 34 patients undergoing LT, blood samples were collected before surgical incision, 15 min after the beginning of the anhepatic phase, and 15 min after arterial revascularization of the graft. Results: Clotting time (CT) was well correlated with prothrombin (PT) ratio and activated partial thromboplastin time (aPTT) ratio. Platelet contribution to clot stiffness (PCS) was correlated with platelets (ρ = 0.82, *p* < 0.001) and fibrinogen contribution clot stiffness (FCS) with fibrinogen (Fg) (ρ = 0.74, *p* < 0.001). CT predicted a PT ratio < 30% with an area under the curve (AUC) of 0.93 (95% CI 0.87–0.98; *p* < 0.001). PCS predicted a platelet count < 50 G/L with an AUC of 0.87 (95% CI 0.76–0.98, *p* < 0.001). FCS predicted a Fg < 1.0, 1.2 or 1.5 g/L, with an AUC of 0.86 (95% CI 0.77–094, *p* < 0.001), 0.82 (95% CI 0.74–0.91, *p* < 0.001) and 0.88 (95% CI 0.82–0.95, *p* < 0.001), respectively. Conclusion: Quantra^®^ provides a rapid assessment of haemostasis during LT.

## 1. Introduction

Liver transplantation (LT) remains a surgery with a high-risk of bleeding, due to cirrhosis, portal hypertension, history of previous surgeries and/or donors’ and grafts’ characteristics. The management of coagulopathy and transfusion is challenging and can be guided with standard laboratory tests and point-of-care devices [1,2,3,4]. Until recently, the two available point-of-care devices in the setting of LT were based on viscoelasticity (viscoelastic tests, VET): thromboelastography (TEG) and rotational thromboelastometry (ROTEM). The implementation of VET in a transfusion algorithm was associated with less blood product transfusion and increased administration of factor concentrates. The impact of these tools on patient outcome remains unknown [5,6].

A newly available point-of-care device, the Quantra^®^ (Stago, Hemosonics, Charlottesville, VA, USA) is based on the sonic estimation of elasticity via resonance (SEER) or sonorheometry, which analyses acoustic radiation force [7]. Briefly, whole blood collected on a citrate tube is activated with different reagents and is submitted to a focused ultrasound pulse. A shear wave is generated, and when a clot begins to form, the sample resonates. The ultrasound pulses generated by the clot vibrations are transmitted and analysed by the device software [8]. The results are available as curves or wheel-shaped dials. The QStat^®^ cartridge allows for the exploration of clotting time (CT), clot stiffness (CS), fibrinogen and platelet contribution to clot stiffness (FCS and PCS) and clot stability to lysis (CSL) [9]. In trauma, and in orthopaedic and cardiac surgery, Quantra^®^ results were well correlated with VET results and standard laboratory results [9,10,11,12,13]. Moreover, in high bleeding-risk cardiac surgery, a Quantra^®^-guided transfusion algorithm was associated with a decrease in transfusion and major bleeding compared to a laboratory-based transfusion algorithm [14].

In the setting of LT, a recent study compared Quantra^®^ to ROTEM^®^ results and showed good correlations [15]. However, the authors did not compare Quantra^®^ results to standard laboratory tests. The main objective of our study was to describe Quantra^®^ parameters during LT and to study their correlations with standard laboratory parameters for their potential benefits in routine practice. The second objective was to determine Quantra^®^ cut-off values for thrombocytopenia, hypofibrinogenemia and coagulation factors’ deficit.

## 2. Materials and Methods

This retrospective monocentric study was registered in the APHP general data processing register (n° 20230704140052) and approved by the Comité d’éthique pour la recherche en Anesthésie-Réanimation (French Committee for Research in Anesthesia and Intensive Care: IRB 00010254, 2023-078, Dr V. Billard). It followed the principles of the Declaration of Helsinki. According to French law, all patients received an information letter but their written consent was not needed.

Quantra^®^ device and QStat^®^ cartridges (Stago, Hemosonics, Charlottesville, VA, USA) were FDA-marked (K213917 and DEN180017, respectively), and complied with all essential requirements of the IVD Directive 98/79/EC. Quantra^®^ was used in compliance with the requirements of the French regulatory standard for the quality of delocalised medical biology ISO/IEC 15189:2022 [16] supervised by COFRAC (accreditation number: N 8-1128, French certification body).

Adult patients undergoing LT at Paul Brousse hospital (APHP, Paris, France) between March and May 2023 were eligible. Grafts came from donors after brain death (DBD), or Maastricht 3 donors after circulatory death (DCD) or living donors for domino LT. Simultaneous kidney–liver transplantations were also included.

Patients’ characteristics and LT’s characteristics were recorded from medical files. Anaesthesia and surgical techniques were standardised. All patients received tranexamic acid (TXA), 1 g intra-venous bolus over 10 min before surgical incision, followed by 1 g over 8 h. To manage coagulopathy, prerequisites were body temperature > 35.5 °C, arterial pH > 7.30, ionised calcaemia > 1 mmol/L and haemoglobin ≥ 8 g/dL (except in case of sickle-cell disease). Blood cell salvaging was used if there were no contra-indications. If needed, preoperative anticoagulation with anti-vitamin K agents was antagonised using prothrombin complex concentrates. Transfusion occurred only in the event of bleeding with the goal to maintain platelets > 30 G/L, fibrinogen (Fg) > 1.5 g/L and prothrombin time (PT) ratio > 30%. In case of bleeding, a Fg concentration < 1.5 g/L led to fibrinogen concentrate administration (25–50 mg/kg), a platelet count < 30 G/L led to platelets transfusion (0.7 × 10^11^/10 kg) and a PT ratio < 30% led to plasma transfusion (≥15 mL/kg). If CSL was below 90%, despite the continuous infusion of TXA, a supplementary 1 g bolus was allowed.

During LT, three blood samples were performed as usual: after induction of general anaesthesia and before administration of TXA (=T1), 15 min after the beginning of the anhepatic phase (=T2) and 15 min after arterial revascularisation of the graft (=T3). At each time point, blood was collected in EDTA tube and sodium citrate 0.109 M (3.2%) tubes (BD Vacutainer^®^). Routine tests (PT, activated partial thromboplastin time (aPTT), fibrinogen, factor II, factor V and D-dimers) were performed using a STA-RMax automate (Stago BioCare, Asnières sur Seine, France) using routine reagents from Stago (NeoPTimal, Automated aPTT, Deficient plasmas, Liatest^®^ D-DiPlus) or from Siemens (Courbevoie, France) (Dade Thrombin Reagent for determining fibrinogen with Clauss method). Blood cell count was performed on EDTA tubes with a XN1500 automate (Sysmex, Villepinte, France). We used QStat^®^ Cartridges for the following Quantra^®^ analyses: clot time (CT), clot stiffness (CS), fibrinogen contribution to clot stiffness (FCS), platelet contribution to clot stiffness (PCS) and CSL (clot stability lysis).

In cardiac surgery and traumatology, correlation coefficients between FCS and Clauss fibrinogen were 0.73 and 0.75, and correlation coefficients between PCS and platelet count 0.48 and 0.66, respectively [11,13]. If one hypothesized a correlation coefficient between FCS and Clauss fibrinogen > 0.7 in the setting of LT, with an α-risk of 0.05, 72 samples would be necessary to provide an 80% power; thus, 24 patients each had three sample sets.

Quantitative data were expressed as median [interquartile range (IQR) 25–75] and qualitative data as numbers (percentages). Spearman rank coefficients were determined, and a principal component analysis (PCA) was performed. The determinants of Quantra^®^ test results were assessed by multiple linear regression using a stepwise model. Receiver-operating characteristic (ROC) curves were calculated to determine the ability of Quantra^®^ parameters to predict coagulation parameters leading to transfusion of platelets, fibrinogen concentrate or fresh frozen plasma. Sensitivity, specificity, positive and negative predictive values (PPV and NPV) were calculated for the best cut-off value. A *p*-value < 0.05 was considered statistically significant. All statistical tests were performed with the XLSTAT V.2023 package (Lumivero, Paris, France).

## 3. Results

### 3.1. Patients and LT Characteristics

From 13 March to 29 May 2023, 37 LTs were performed in Paul Brousse Hospital. One was excluded from the analysis for technical reasons, and two were because they were simultaneous heart–liver transplantations. Patients, donors and LT characteristics are presented in Table 1. Patients were mostly men, with a median age of 60.5 years and a median MELD score of 20 [12–32]. The main liver disease leading to LT was alcoholic cirrhosis (*n* = 20, 58.8%). Patients suffering from cirrhosis mainly had a Child–Pugh score C (*n* = 18, 52.9%) and a median MELD score of 21 [16–33]. Hepato-cellular carcinoma (HCC) was the main cause for LT in 7 (20.6%) patients, but a total of 16 patients (47.1%) had HCC. Two LT were for redo surgeries (5.9%). Two patients had combined liver–kidney transplantations (5.9%). Four patients were on long-term aspirin therapy, one had a long-term anti-vitamin K agent and one suffered from dysfibrinogenemia.

Bleeding and transfusion data are presented in Table 2. Median preoperative bleeding was 1500 [1025–2525] mL. Cell salvage was used for 20 procedures and allowed the re-transfusion of 262 [179–567] mL. Twenty patients (58.8%) needed a transfusion of one or more blood labile product and seven (20.6%) needed an administration of fibrinogen concentrate. The amount of plasma transfused was slightly below the preconized dose in the protocol, whereas the amount of fibrinogen administered was in the target. Five patients (14.7%) received platelet transfusion. Only two patients received more than 2 g of TXA.

The results of laboratory tests and Quantra^®^ tests are presented in Table 3. During the LT, platelet count, PT ratio, factors II and V, fibrinogen, CS, PCS and FCS decreased, whereas aPTT ratio and CT increased. D-dimers and CSL remained quite stable through the procedure.

### 3.2. Principal Component Analysis

**Figure 1:**
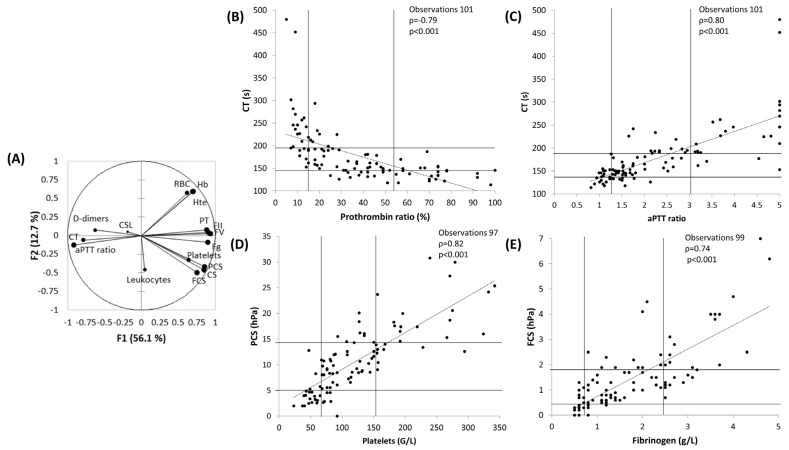
(**A**) Principal component analysis (PCA) including all biological parameters from all samples. (**B**) Scatter plot depicting CT versus PT ratio. (**C**) Scatter plot depicting CT versus aPTT ratio. (**D**) Scatter plot depicting PCS versus platelet count. (**E**) Scatter plot depicting FCS versus fibrinogen. Horizontal and vertical lines represent the quartiles 25 and 75 of the parameter on the vertical and horizontal axis, respectively. Abbreviations: aPTT, activated partial thromboplastin time; CS, clot stiffness; CSL, clot stability lysis; FII, factor II; FV, factor V; FCS, fibrinogen contribution to clot stiffness; Fg, fibrinogen; Hb, haemoglobin; hPa, hectopascal; Hte, haematocrit; PCS, platelet contribution to clot stiffness; PT, prothrombin time ratio; RBC, red blood cells.

In the PCA including the results of all the biological parameters (laboratory and Quantra^®^) presented Figure 1A, 56.1% of the variability of the scatter plot is represented on the horizontal axis (F1); we can call that the “coagulation axis”. The vertical axis represents 12.7% of the variability. The larger the dot, the higher the squared cosines, reflecting the representation quality of a variable on the PCA axis. Moreover, dots are not interpretable if they are too close to the centre. Thus, no conclusion can be drawn for CSL, D-dimers and leukocytes. Furthermore, the sharper the angle between two variables, the more correlated they are. Figure 1B–E represent the scatter plots between the laboratory and the Quantra^®^ parameters, which are more correlated according to Spearman rank correlation.

CT was correlated with PT ratio (ρ = −0.79, *p* < 0.001) and aPTT ratio (ρ = 0.80, *p* < 0.001). PCS was correlated with platelets (ρ = 0.82, *p* < 0.001) and FCS with Fg (ρ = 0.74, *p* < 0.001). These correlations were stable whatever the sampling time. For example, Spearman rank correlations between FCS and Fg were 0.73 at T1 (*p* < 0.001), 0.66 at T2 (*p* < 0.001) and 0.67 at T3 (*p* < 0.001).

As Fg administration was not performed per-operatively by all the liver transplant teams, we also performed the analyses excluding the data from the seven patients who received fibrinogen perioperatively. Spearman rank coefficients remained good and in the same range as those obtained with the whole data set: between CT and PT ratio (ρ = −0.78, *p* < 0.001), CT and aPTT ratio (ρ = 0.80, *p* < 0.001), PCS and platelets (ρ = 0.81, *p* < 0.001) and FCS and Fg (ρ = 0.68, *p* < 0.001).

### 3.3. Multiple Linear Regression

**Figure 2:**
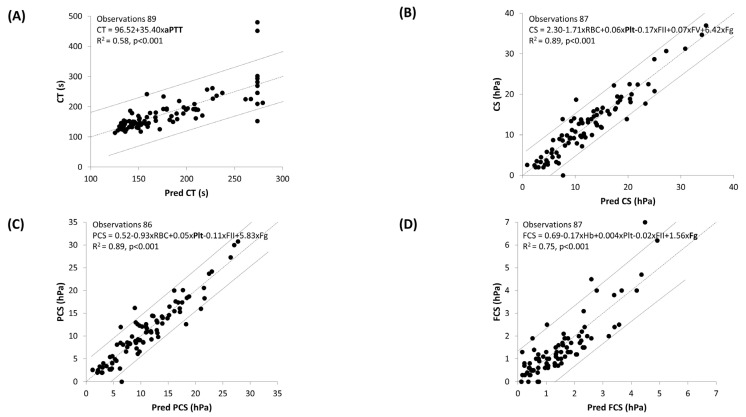
Multiple linear regressions by stepwise model for (**A**) CT, (**B**) CS, (**C**) PCS and (**D**) FCS. The dark dotted line represents the model, and the grey lines the 95% confidence interval. In the model equations, the parameter in bold has the most weight in the regression. Abbreviations: CS, clot stiffness; CT, clotting time; FII, factor II; FV, factor V; FCS, fibrinogen contribution to clot stiffness; Fg, fibrinogen; Hb, haemoglobin; hPa, hectopascal; PCS, platelet contribution to clot stiffness; Plt, platelets; Pred, predicted; RBC, red blood cells.

To decipher the determinants of Quantra^®^ test results, we conducted multiple linear regressions using a stepwise model (Figure 2). As seen in Figure 2A, aPTT explained 58% of the variability of CT. For CS and PCS (Figure 2A and Figure 2B, respectively), 89% of the variability of the parameters can be explained by a model including RBC, platelets, FII, FV and Fg, with platelets being the most contributing factor. As for FCS, a model including Hb, platelets, FII and Fg (the most contributing factor) explained 75% of its variability (Figure 2D). No linear regression could be generated for CSL.

### 3.4. ROC Curves Analysis

**Figure 3:**
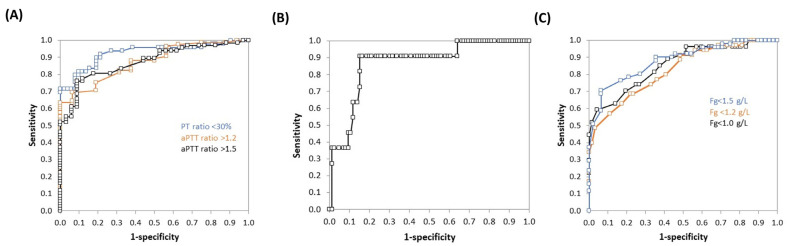
Receiving operator characteristic curve analyses. (**A**) Ability of CT to detect PT ratio < 30%, aPTT ratio > 1.2 or aPTT ratio > 1.5. (**B**) Ability of PCS to detect platelet count < 50 G/L. (**C**) Ability of FCS to detect Fg < 1.0 or 1.2 or 1.5 g/L. The analysis was based on 101 observations for CT, 97 for PCS and 99 for FCS. Abbreviations: aPTT, activated partial thromboplastin time; CS, clot stiffness; CT, clotting time; FCS, fibrinogen contribution to clot stiffness; Fg, fibrinogen; PCS, platelet contribution to clot stiffness; PT, prothrombin time.

To refine the place of Quantra^®^ in our transfusion algorithm, we conducted ROC curves analyses to determine the best cut-off value of CT to predict PT ratio < 30% or aPTT > 1.2 or 1.5, of PCS to predict platelet count < 50 G/L and of FCS to predict Fg < 1.0, 1.2 or 1.5 g/L (Figure 3 and Table 4). CT predicted a PT ratio < 30% with an AUC of 0.93 (95% CI 0.87–0.98; *p* < 0.001), and this was the highest AUC of the Quantra^®^ values (Figure 3A). The best cut-off value was 166 s, with a sensitivity of 0.82 (95% CI 0.68–0.90), a specificity of 0.90 (95% CI 0.79–0.96), a PPV of 0.89 and a NPV of 0.84. PCS predicted a platelet count < 50 G/L with an AUC of 0.87 (95% CI 0.76–0.98, *p* < 0.001). The best cut-off value was 5.4 hPa, but with a low PPV at 0.44 (Figure 3B). FCS predicted a Fg < 1.0, 1.2 or 1.5 g/L with an AUC of 0.86 (95% CI 0.77–094, *p* < 0.001), 0.82 (95% CI 0.74–0.91, *p* < 0.001) and 0.88 (95% CI 0.82–0.95, *p* < 0.001), respectively (Figure 3C). The best cut-off values were 0.6, 0.9 and 1.0 hPa, respectively.

The sensitivity analysis performed without the data from the seven patients who received Fg found different AUC and thresholds values, as shown in Table 5.

**Figure 4:**
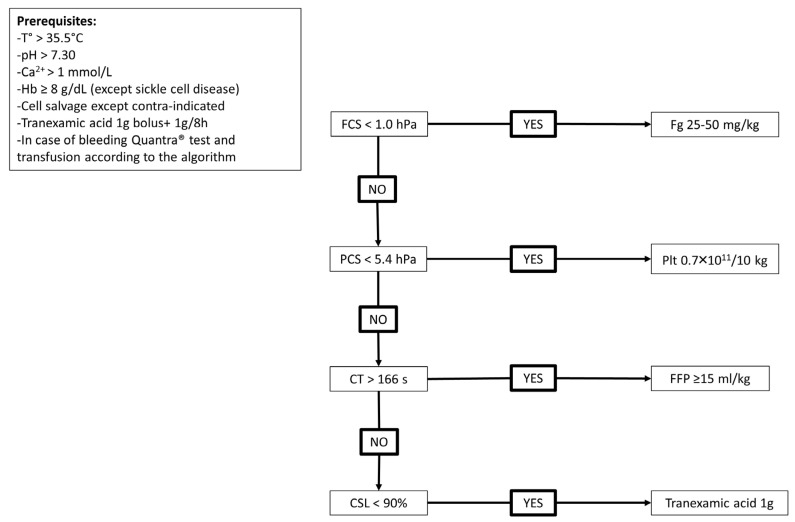
Proposition of transfusion algorithm including Quantra^®^ results. Abbreviations: CSL, clot stability lysis; CT, clotting time; FCS, fibrinogen contribution to clot stiffness; FFP, fresh frozen plasma; Fg, fibrinogen; Hb, haemoglobin; hPa, hectopascal; PCS, platelet contribution to clot stiffness; Plt, platelets; T°, body core temperature.

According to these results, we propose a transfusion algorithm including Quantra^®^ parameters, which should only be used in case of bleeding (Figure 4).

## 4. Discussion

In 34 patients benefiting from LT, Quantra^®^ parameters correlated well with standard laboratory results, with a Spearman rank coefficient ranging from 0.74 between FCS and Fg to 0.82 between PCS and platelet count. These results are in line with those found in the context of cardiac or orthopaedic surgery [11,17,18], and are slightly better than those observed in trauma [13].

CSL and D-dimers were poorly correlated. The first blood sampling was performed before the bolus of TXA, and the two others while TXA was continuously infused. The correlation remains poor even when focusing on the first sample set. Flores et al. have shown good concordance between ROTEM^®^ and Quantra^®^ for fibrinolysis [15], but one must keep in mind the poor sensitivity of ROTEM^®^ for diagnosing hyperfibrinolysis [19]. Moreover, D-dimers may not be a good surrogate marker for hyperfibrinolysis when TXA is continuously infused [20]. To test the ability of Quantra^®^ to detect hyperfibrinolysis, specific tests (dosages of t-PA and PAI-1 activity) and a global fibrinolysis capacity assay are needed, or the euglobulin clot lysis time, which is insensitive to the presence of TXA [21].

As no “normal” values for Quantra^®^ or laboratory parameters have been determined in cirrhotic patients, we did not conduct a concordance analysis between these parameters and the results of standard laboratory tests, and we chose to represent the interquartile range for each parameter (Figure 1). In the multiple linear regression (Figure 2), the analysis of CT was impaired by the fact that the laboratory did not provide an aPTT ratio result above five (upper limit of detection). When removing the 11 observations with an aPTT ≥ 5, the equation of the model became CT = 49.8 − 10.2 × Hb + 4.98 × Hte + 32.46 × aPTT + 0.002 × D-dimers (R^2^ = 0.72, *p* < 0.001), and the aPTT remained the parameter with the most weight in the equation. Platelet count was the most important factor in the regression equation for predicted CS and PCS, whereas Fg had the most weight in the regression equation for predicted FCS.

ROC curve results were good to excellent, with AUC ranging from 0.82 (ability of FCS to detect hypofibrinogenemia < 1.2 g/L), to 0.93 (ability of CT to detect PT ratio < 30%). These AUC are in line with those described in major surgery or in trauma [13,17]. We explored the ability of CT to detect PT ratio < 30% and aPTT ratio > 1.2 or 1.5, and the best conjunction of AUC, sensitivity, specificity, PPV and NPV is for PT ratio < 30%. Despite our haemostatic objective of platelets > 30 G/L in case of bleeding, we did not perform ROC curve analysis for platelets < 30 or 40 G/L, because only one and three cases had such low platelet counts, respectively, whereas eleven cases had platelets < 50 G/L. It is noteworthy that for this parameter, the NPV is higher than the PPV. Finally, we tested the ability of FCS to predict various thresholds of Fg, as there is no consensus in the literature as to the best objective in the case of bleeding during LT (between 1.0 and 1.5 g/L). However, low preoperative Fg is associated with higher perioperative bleeding and need for transfusion, and the threshold seems to be between 1 and 2 g/L [22,23]. Based on these ROC curves analyses, we propose a transfusion algorithm including the Quantra^®^ results. AUC for CT and PCS were better than AUC for FCS, but platelets infusion efficiency is of short duration in cirrhotic patients with splenomegaly [24,25] and high volumes of plasma are needed to correct coagulation deficiency, with a risk of worsening portal hypertension and bleeding [4].

As pre- or per-operative administration of Fg is not performed by all LT teams, we conducted a sensitivity analysis excluding the data from the seven patients who received Fg. Spearman rank coefficients between Quantra^®^ parameters and laboratory parameters remained good and in the same range as those obtained with the whole data set. In contrast, ROC curve analyses showed different AUC and thresholds for FCS to detect hypofibrinogenemia below 1.0, 1.2 or 1.5 g/L. This can be explained by the lowest number of cases, as. in our protocol, bleeding patients with hypofibrinogenemia were eligible for Fg administration, but also by the influence of Fg concentrate administration on the correlation between FCS and Fg Clauss, as experienced with ROTEM^®^ in cardiac surgery [26]. However, the best AUC is still for the detection of Fg < 1.5 g/L, with the best FCS cut-off value of 1.0 hPa, and our proposition of transfusion algorithm remains unchanged.

As other viscoelastic tests, Quantra^®^ has advantages (whole blood test, global vision of coagulation) and limitations (insensitivity to Von Willebrand factor and to the anticoagulant protein C system). This must be kept in mind when exploring cirrhotic patients’ coagulation [27].

Our study had both strengths and limitations. Although it is retrospective and monocentric and our results must be confirmed in prospective multicentric studies, this study clearly shows the strong correlation of SEER-derived haemostasis variables with classical laboratory values. As it is observational, the impact of a Quantra^®^-guided transfusion algorithm on clinically relevant outcomes (per-operative bleeding and transfusion, intensive care unit and hospital length of stay, haemorrhagic and thrombotic complications) remains to be determined. We did not measure the time between blood sampling and results, but in a previous study, this delay was significantly shorter for Quantra^®^ tests than for laboratory tests [13]. This tool provides a rapid assessment of haemostasis during haemorrhage, and prospective randomised controlled studies may determine its impact on patient care. The algorithm we propose here has yet to be validated in large studies.

In conclusion, in this series of 34 LTs in an expert centre, we demonstrated a good correlation of the values of coagulation parameters between the technology of the point-of-care Quantra^®^ device and those of laboratory tests used to currently monitor fresh frozen plasma, platelets and fibrinogen concentrate transfusions. Because of the time saved with each transplantation by avoiding the time spent on transporting the collection tubes and their centrifugation within the on-call laboratory, the Quantra^®^ solution represents an obvious advantage in the care of the graft recipient.

## Figures and Tables

**Table 1:** Patients’ and liver transplantations’ characteristics.

Characteristics	Values
**Patients (*n* = 34)**	
Gender M/F, *n* (%)	27 (79.4)/7 (20.6)
Age, years	60.5 [51.2–63.8]
Height, m	1.71 [1.65–1.78]
Weight, kg	86.0 [78.0–93.2]
BMI, kg/m^2^	29.4 [25.2–31.7]
Liver disease leading to LT, *n* (%)	
Alcoholic cirrhosis	20 (58.8)
HCC	7 (20.6)
Acute hepatitis	5 (14.7)
Amyloid neuropathy	1 (2.9)
Hepatic metastasis	1 (2.9)
MELD score	20 [12–32]
Child–Pugh score A/B/C/NA	8 (23.5)/5 (14.7)/18 (52.9)/3 (8.8)
**Donors (*n* = 34)**	
Age (years) Type of donors: DBD/M3 DCD/LDD	58 [48–71] 32 (94.2)/1 (2.9)/1(2.9)
**Liver transplantations (*n* = 34)**	
Surgical time, min	424 [373–487]
Cold ischemia time, min	359 [307–426]
Warm ischemia time, min	30 [26–37]
Type of caval anastomosis: 3 veins piggyback technique/side to side/caval replacement	27 (79.4)/2 (5.9)/5 (14.7)

Note: Data are expressed as median [interquartile range] or number (%). Abbreviations: BMI, body mass index; DBD, donor after brain death; DCD, donor after circulatory death; HCC, hepato-cellular carcinoma; LDD, living donor domino; M3, Maastricht 3; MELD, model for end-stage liver disease; NA, non-appropriate.

**Table 2:** Bleeding and transfusion.

Characteristics Number of Patients Concerned (%)	Values
Bleeding, mL	1500 [1025–2525]
Re-transfused blood volume from cell salvage (mL) *N* = 20 (58.8%)	262 [179–567]
Blood labile products transfusion, *n* (%)	20 (58.8)
PRBC, *n* *N* = 18 (52.9%)	4 [3–7]
FFP, *n* *N* = 11 (32.4%)	3 [2–8]
FFP, mL/kg *N* = 11 (32.4%)	10.6 [7.0–20.7]
Platelet units, *n* *N* = 5 (14.7%)	1 [1–2]
Fg concentrate, g *N* = 7 (20.6%)	3.0 [2.5–3.5]
Fg concentrate, mg/kg *N* = 7 (20.6%)	33 [24–38]
Tranexamic acid, g *N* = 34 (100.0%)	1.8 [1.7–2.0]
Tranexamic acid, mg/kg *N* = 34 (100.0%)	21.8 [19.1–24.6]

Note: The amounts of transfusion are detailed only for transfused patients. Data are expressed as median [interquartile range] or number (%). Abbreviations: FFP, fresh frozen plasma; Fg, fibrinogen; PRBC, pack of red blood cells.

**Table 3:** Haemostatic testing.

Variables (Normal Values for Healthy Controls)	All	T1	T2	T3
**Laboratory results**				
Leukocytes, G/L (4.0–10.0)	7.3 [5.5–9.0]	6.5 [4.7–8.2]	6.4 [5.4–9.7]	8.0 [6.5–9.7]
Red blood cells, T/L (3.8–5.0)	3.0 [2.5–3.8]	3.6 [2.5–4.2]	3.0 [2.6–3.5]	2.8 [2.3–3.2]
Haemoglobin, g/dL (12.0–16.0)	9.2 [7.9–11.4]	10.8 [7.9–12.8]	9.2 [8.2–11.2]	8.3 [7.5–10.1]
Haematocrit, % (34.0–45.0)	27.0 [23.4–33.3]	31.2 [23.2–37.0]	27.0 [24.6–32.7]	24.6 [22.2–29.5]
Platelets, G/L (150.0–400.0)	107.5 [70.2–155.8]	100.5 [67.5–156.8]	116.0 [77.0–163.5]	85.5 [70.5–140.2]
Prothrombin ratio, % (>70)	32 [15–52]	42 [20–70]	32 [18–50]	21 [13–40]
Factor II, % (60–140)	27 [12–51]	38 [25–69]	28 [14–52]	13 [10–34]
Factor V, % (60–140)	28 [17–51]	41 [27–73]	29 [18–47]	17 [11–33]
aPTT ratio (<1.20)	1.76 [1.34–3.08]	1.48 [1.17–1.97]	1.74 [1.45–2.78]	2.80 [1.66–4.95]
Fibrinogen, g/L (1.5–4.0)	1.4 [0.8–2.5]	2.1 [1.2–2.8]	1.4 [0.9–2.4]	1.1 [0.8–1.8]
D-dimers, ng FEU/mL (<500)	2085 [1111–3474]	2235 [669–7085]	1840 [1128–3290]	2443 [1436–3228]
**Quantra^®^ results**				
CT, s (103–153)	161 [144–194]	150 [136–180]	152 [138–164]	194 [177–226]
CS, hPa (13.0–33.2)	11.2 [6.2–16.2]	13.6 [7.2–18.1]	12.4 [8.0–16.7]	9.0 [5.4–12.0]
PCS, hPa (11.9–29.8)	9.9 [5.6–14.4]	12.0 [8.6–16.1]	11.3 [6.6–14.6]	8.1 [4.6–10.7]
FCS, hPa (1.0–3.7)	1.1 [0.6–1.9]	1.6 [0.8–2.2]	1.2 [0.7–1.7]	1.0 [0.5–1.2]
CSL, % (>92)	99 [98–100]	100 [98–100]	99 [98–100]	100 [98–100]

Note: Data are expressed as median [interquartile range]. T1 = after induction of general anaesthesia and before administration of tranexamic acid, T2 = 15 min after the beginning of the anhepatic phase, T3 = 15 min after arterial revascularisation of the graft. Abbreviations: aPTT, activated partial thromboplastin time; CS, clot stiffness; CT, clotting time; CSL, clot stability lysis; FCS, fibrinogen contribution to clot stiffness; PCS, platelet contribution to clot stiffness.

**Table 4:** Synthesis of receiving operator characteristics curves analyses.

Parameters	AUC	Best Cut-Off	Sensitivity	Specificity	PPV	NPV
Ability of CT to predict						
PT ratio < 30%	0.93 (0.87–0.98) *	166	0.82 (0.68–0.90)	0.90 (0.79–0.96)	0.89	0.84
aPTT ratio > 1.2	0.87 (0.80–0.95) *	155	0.64 (0.53–0.73)	1.00 (0.77–1.00)	1.00	0.34
aPTT ratio > 1.5	0.87 (0.81–0.94) *	155	0.76 (0.65–0.85)	0.92 (0.76–0.98)	0.94	0.66
Ability of PCS to predict						
Plt count < 50 G/L	0.87 (0.76–0.98) *	5.4	0.91 (0.59–1.00)	0.85 (0.76–0.91)	0.44	0.99
Ability of FCS to predict						
Fg < 1.0 g/L	0.86 (0.77–0.94) *	0.6	0.59 (0.41–0.75)	0.96 (0.88–0.99)	0.84	0.86
Fg < 1.2 g/L	0.82 (0.74–0.91) *	0.9	0.69 (0.52–0.81)	0.78 (0.66–0.87)	0.63	0.82
Fg < 1.5 g/L	0.88 (0.82–0.95) *	1.0	0.71 (0.57–0.81)	0.94 (0.82–0.98)	0.92	0.75

Note: The analysis was based on 101 observations for CT, 97 for PCS and 99 for FCS. AUC, sensitivity, specificity, PPV and NPV are given with 95% confidence intervals. Abbreviations: aPTT, activated partial thromboplastin time; AUC, area under the curve; CT, clotting time; FCS, fibrinogen contribution to clot stiffness; Fg, fibrinogen; NPV, negative predictive value; PCS, platelet contribution to clot stiffness; Plt, platelet; PT, prothrombin; PPV, positive predictive value. * *p* < 0.001.

**Table 5:** Receiving operator characteristics curves analysis in patients not receiving Fg.

Parameters	AUC	Best Cut-Off	Sensitivity	Specificity	PPV	NPV
Ability of FCS to predict						
Fg < 1.0 g/L	0.81 (0.68–0.93) *	1.3	0.87 (0.61–0.97)	0.62 (0.50–0.73)	0.35	0.95
Fg < 1.2 g/L	0.80 (0.70–0.91) *	1.5	0.91 (0.71–0.98)	0.55 (0.42–0.68)	0.44	0.94
Fg < 1.5 g/L	0.85 (0.76–0.94) *	1.0	0.61 (0.44–0.75)	0.96 (0.84–0.99)	0.91	0.77

Note: The analysis was based on 78 observations. AUC, sensitivity, specificity, PPV and NPV are given with 95% confidence intervals. Abbreviations: AUC, area under the curve; FCS, fibrinogen contribution to clot stiffness; Fg, fibrinogen; NPV, negative predictive value; PPV, positive predictive value. * *p* < 0.001.

## Data Availability

The data presented in this study are available on request from the corresponding author.
